# Application of a Novel S3 Nanowire Gas Sensor Device in Parallel with GC-MS for the Identification of Rind Percentage of Grated Parmigiano Reggiano

**DOI:** 10.3390/s18051617

**Published:** 2018-05-18

**Authors:** Marco Abbatangelo, Estefanía Núñez-Carmona, Veronica Sberveglieri, Dario Zappa, Elisabetta Comini, Giorgio Sberveglieri

**Affiliations:** 1 Department of Information Engineering, University of Brescia, Via Branze 38, 25123 Brescia, Italy; e.nunezcarmona@unibs.it (E.N.-C); dario.zappa@unibs.it (D.Z.); elisabetta.comini@unibs.it (E.C.); giorgio.sberveglieri@unibs.it (G.S.); 2 CNR-IBBR, Institute of Biosciences and Bioresources, Via Madonna del Piano 10, 50019 Sesto Fiorentino (FI), Italy; veronica.sberveglieri@ibbr.cnr.it; 3 NANO SENSOR SYSTEMS S.r.l., Via Branze 38, 25123 Brescia, Italy

**Keywords:** electronic nose, nanowire gas sensors, food quality control, Parmigiano Reggiano, multivariate data analysis, artificial neural network

## Abstract

Parmigiano Reggiano cheese is one of the most appreciated and consumed foods worldwide, especially in Italy, for its high content of nutrients and taste. However, these characteristics make this product subject to counterfeiting in different forms. In this study, a novel method based on an electronic nose has been developed to investigate the potentiality of this tool to distinguish rind percentages in grated Parmigiano Reggiano packages that should be lower than 18%. Different samples, in terms of percentage, seasoning and rind working process, were considered to tackle the problem at 360°. In parallel, GC-MS technique was used to give a name to the compounds that characterize Parmigiano and to relate them to sensors responses. Data analysis consisted of two stages: Multivariate analysis (PLS) and classification made in a hierarchical way with PLS-DA ad ANNs. Results were promising, in terms of correct classification of the samples. The correct classification rate (%) was higher for ANNs than PLS-DA, with correct identification approaching 100 percent.

## 1. Introduction

Parmigiano Reggiano (PR) cheese is among the most typical Italian foods and one of the oldest traditional cheeses produced in Europe. It is also the most important Protected Designation of Origin (PDO) Italian cheese in terms of commercial importance [1]. Its production is regulated by the Parmigiano Reggiano Cheese Consortium (CFPR). According to European Regulation 510/2006, this designation can be exclusively assigned to the cheese only when it is made with a traditional established production technology in a restricted area of Italy (provinces of Parma, Reggio Emilia, Modena, Mantova and Bologna) from milk produced in the same area [2].

PR can be found on the market in different forms. It can be portioned or grated and cannot be subjected to any treatment like lyophilization, drying and freezing [3]. All the procedures, which must be followed to obtain the original PR, make this cheese a high-value product. This leads to a final product that has various nutritional properties: its dry weight is mostly composed of proteins and lipids, it is lactose- and galactose-free and it is rich in organic acids, such as lactic acid, acetic acid, propionic and butyric acids [4]. The semi-fat composition, due to natural creaming of skimmed unpasteurized milk [5], is produced by cattle that consume only locally grown forage because supply of silage and fermented feeds is not permitted [6].

For these reasons, PR has a high cost when compared to similar hard cheeses. This encourages the appearance on the market of counterfeited products that bear the PR brand at a lower price. The rate of fraud is estimated to be between 20% and 40%, the latter predominantly in the grated form [7].

As established in the procedural guideline, grated PR cheese must follow some technical and technological parameters: moisture no less than 25% and no more than 35%, at least 12 months of ripening, rind percentage compared to pulp not over 18% (by weight), typical amino-acid composition of the cheese, absence of additives, not crumbly in aspect and with homogeneous particles that have a diameter inferior to 0.5 mm and do not exceed 25% [8].

In order to determine if a PR cheese package conforms to the rules, the aromatic profile of grated PR can be analyzed thanks to the volatile organic compounds (VOCs). VOCs of various dairy products have received a great deal of attention in recent years. Until now, about 600 volatile compounds have been identified for cheese [9]. However, only a small part of these compounds is responsible for cheese flavor [10]. Cheese aroma is considered the result of the equilibrium between various VOCs that, separately, do not reflect the overall odor [11]. Hydrocarbons, alcohols, aldehydes, ketones, esters and lactones were the major classes of compounds found in the neutral fraction of cheese [12].

In this work, an electronic nose has been used in order to analyze rind percentage in grated PR cheese through emitted VOCs. In recent years, this kind of device has received considerable attention for its potentialities; it has been applied in various fields, such as environment [13,14,15,16,17], health [18,19,20,21,22] and food, with excellent results [23,24,25]. Regarding food applications, some examples of electronic nose applications are the detection of microorganisms in tomato sauce [26] and of different molds in coffee [27], the determination of the shelf life of milk [28], the detection of additives in fruit juices [29], the identification of fruit [30] and the discrimination of cheese varieties [31,32]. These few examples show how e-noses have the potential to be used in different ways to assess food quality and identity.

Placed side by side with e-nose analysis, Gas Chromatography coupled with Solid Phase Micro Extraction (SPME) was used. SPME has received a great deal of attention in the literature to find VOCs that characterize food matrices. Many foods have been studied, including dairy products, such as milk [33], butter [34] and cheese [35,36].

The aim of this preliminary research is to determine the rind percentage of the sample under analysis with an innovative and rapid methodology, in order to identify frauds and therefore have available an affordable and reliable instrument to reduce them, thanks to the different VOCs, in terms of presence and amount, between products. 

## 2. Materials and Methods

### 2.1. Samples Preparation and Experimental Design

Analyzed samples were packaged under vacuum at the headquarters of CFPR. They came from two different ripening stages: 12 and 24 months. For each of these, five different combinations of pulp-rind were prepared (expressed in rind percentage): 0%, 18%, 26%, 45% and 100%. In addition, two kinds of rind working processes were considered: washed-rind (WR) and scraped-rind (SR). The only exceptions are represented by 0% samples, for which only the 24-month ripening was taken into account, and 100% samples, for which there is one for WR, and one for SR, that corresponds to 24-month and 12-month seasoning, respectively. For each sample, 14 replicas were arranged for a total of 210 (14 replicas × 15 samples).

Samples were stored at 4 °C until the moment when they were prepared for the analysis. The amount of 2 g of grated cheese was positioned in 20 mL glass headspace vials and sealed by a metal cap with a PTFE-silicon membrane, crimped with an aluminum crimp.

### 2.2. GC-MS Analysis

The Gas Chromatograph (GC) used during the analyses was a Shimadzu GC2010 PLUS (Kyoto, KYT, Japan), equipped with a Shimadzu single quadrupole Mass Spectometer (MS) MS-QP2010 Ultra (Kyoto, KYT, Japan) and an autosampler HT280T (HTA S.r.l., Brescia, Italy). The GC-MS analysis was coupled with the Solid-Phase Micro Extraction (SPME) method in order to find the most significant VOCs, which allows for recognition of the different kinds of cheeses.

The fiber used for the adsorption of volatiles was a DVB/CAR/PDMS-50/30 µm (Supelco Co. Bellefonte, PA, USA). The fiber was exposed to the headspace of the vials after heating the samples in the HT280T oven, thermostatically regulated at 50 °C for 15 min, with the aim of creating the headspace equilibrium. The length of the fiber in the headspace was kept constant. Desorption of volatiles took place in the injector of the GC-MS for 6 min at 250 °C.

The gas chromatograph was operated in the direct mode throughout the run, with the mass spectrometer in electron ionization (EI) mode (70 eV). GC separation was performed on a MEGA-WAX capillary column (30 m × 0.25 mm × 0.25 μm, Agilent Technologies, Santa Clara, CA, USA). Ultrapure helium (99.99%) was used as the carrier gas, at the constant flow rate of 1.3 mL/min. The following GC oven temperature programming was applied. At the beginning, the column was held at 40 °C for 8 min, and then raised from 40 to 190 °C at 4 °C/min; then, the temperature was maintained at 190 °C for 5 min. Next, the temperature was raised from 190 °C to 210 °C, with a rate of 5 °C/min; finally, 210 °C was maintained for 5 min.

The GC-MS interface was kept at 200 °C. The mass spectra were collected over the range of 45 to 500 *m*/*z* in the Total Ion Current (TIC) mode, with scan intervals at 0.3 s. The identification of the volatile compounds was carried out using the NIST11 and the FFNSC2 libraries of mass spectra.

The described parameters have been optimized for this specific application. Each sample was analyzed one time.

### 2.3. S3 Analysis

The innovative Small Sensors System S3 device used in the present work has been completely designed and constructed at SENSOR Laboratory (University of Brescia, Italy) in collaboration with NASYS S.r.l., a spin-off of the University of Brescia. The tool comprises a metal oxide (MOX) gas sensors array, flow sensors, temperature and humidity sensors, fluidodinamic system, electronic control system. In particular, the sensors used in this study are 8 MOX gas sensors. Three of them are nanowires of MOX, as presented in References [37,38]. Two of them are tin oxides nanowires sensors, both grown by means of the Vapor Liquid Solid technique [39], using a gold catalyst on the alumina substrate and functionalizing one of them with gold clusters; the third sensor has an active layer of copper oxide nanowires. The working temperature is 350 °C, 350 °C and 400 °C, respectively. The other three sensors are prepared with Rheotaxial Growth and Thermal Oxidation (RGTO) thin film technology; one is tin oxide functionalized with gold clusters (working at 400 °C), while the other two are pure tin oxide (working at 300 °C and at 400 °C, respectively).

The last two are commercial MOX sensors produced by Figaro Engineering Inc. (Osaka, Japan). In particular, they are the TGS2611 and TGS2602, which are sensitive to natural gases and odorous gases like ammonia, respectively, according to the datasheet of the company. Commercial sensors have been mounted on our e-nose in order to evaluate the performances of nanowire sensors. Details of S3 sensors made at SENSOR Laboratory are summarized in Table 1. Response to 5 ppm of ethanol, selectivity (response ethanol/response carbon monoxide) and limit of detection (LOD) of ethanol are also included.

**Figure 1:**
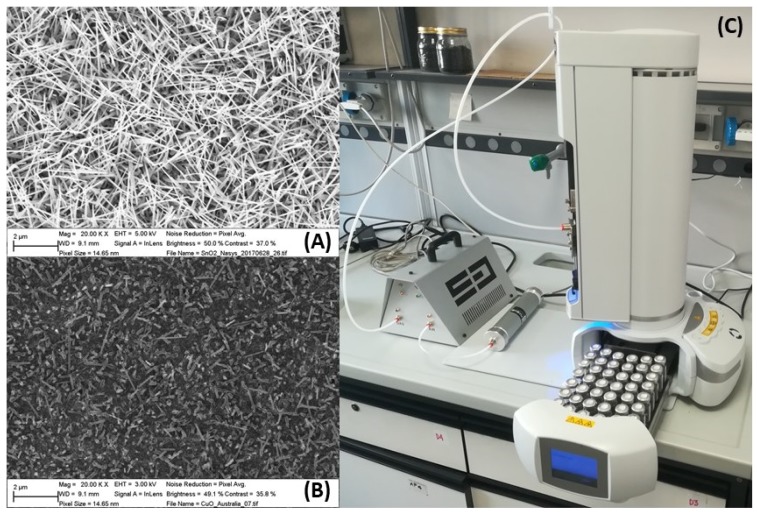
(**A**) SEM image of SnO_2_ nanowires. (**B**) SEM image of CuO nanowires. (**C**) Experimental setup formed by S3 and autosampler.

The MOX nanowires are gas sensors with a high sensitivity to a broad range of chemicals; they exhibit physical properties that are significantly different from their polycrystalline counterpart. The nanowires have a high degree of crystallinity, atomically-sharp terminations and an extraordinary length-to-width ratio, resulting in enhanced sensing capability as well as long-term material stability for prolonged operation. In addition, the three-dimensional network formed by the nanowires increases the adsorption surface and the catalytic activity, improving the response and decreasing the instrument threshold [40]. SnO_2_ and CuO nanowires structures obtained from SEM are shown in Figure 1A,B, respectively.

S3 analyzes the head space (HS), i.e., the volatile fraction of the samples formed when the equilibrium of the solid–liquid phase and the vapor phase of all volatile compounds is reached. The creation of the HS depends on the test substance (vapor pressure) and the conditioning temperature of the sample. The compounds are extracted at the equilibrium point between the solid phase and the vapor in a dynamic head space. This characteristic allows for a non-destructive samples analysis. In this case, the sensor base line is obtained from the air of the surrounding environment; no gas cylinder of chromatographic air is required (an essential feature that makes it a portable instrument). The environmental air was filtered using a small metal cylinder (21.5 cm in length, 5 cm in diameter) filled with activated carbons.

The volatile fraction is then aspirated and transported to the sensor chamber to be analyzed. In order to avoid any influence of the surrounding environment to the sensor response, the chamber has been thermostated and isolated. To prevent the absorption of volatile substances that could be released during subsequent analysis, the chamber and the connection between the elements’ tires are made using steel. The air is flown into the sensor chamber using a pump through a needle valve. This is used to adjust the total airflow, which is measured by a flowmeter downstream from the pump.

The instrument was also provided with the auto-sampler head space system HT2010H, supporting a 42-loading-sites carousel and a shaking oven to equilibrate the sample head space. The vials were placed in a randomized mode into the carousel. Each vial was incubated at 50 °C for 5 min in the auto-sampler oven and shaken for 1 min during the incubation. The sample head space was then extracted from the vial in the dynamic head space path and released into the carried flow (80 sccm). Figure 1C shows the experimental setup (S3 and auto-sampler with its carousel filled with vials).

The analysis timeline can be divided into three different steps for a duration of 420 s (7 min) per sample, which are preceded by a warm-up step that allows for the achievement of the base line for the entire system:

- *Injection*: the sample HS is flowed in the sensor chamber for 60 s (actual analysis time); then, for 30 s, environmental air flows through the same tube to clean it from any residual VOCs;
- *Restore*: when the *injection* period is finished, the filtered air is flowed into the sensors camber. During this time (330 s), the sensors restore the original condition of the base line.

Thanks to the processor integrated in the S3 instrument, the frequency at which the equipment works is equal to 1 Hz.

### 2.4. Data Analysis

Data analysis was performed using MATLAB^®^ R2015a software (MathWorks, USA). First of all, sensors responses in terms of resistance (Ω) were normalized when compared to the first value of the acquisition (R_0_). For all the sensors, the difference between the first value and the minimum value during the analysis time was calculated. Hence, the dataset was composed of ΔR/R_0_ parameters.

In the second step, the normal distribution of the variables was checked using the Jarque-Bera (JB) test, with a significance level equal to 0.05 chosen. This test is a goodness-of-fit test of whether sample data have the skewness and kurtosis matching a normal distribution. The null hypothesis is a joint hypothesis from both the skewness and the excess kurtosis being zero.

Based on the test result, Partial Least Squares (PLS) method was used both to view how the groups of samples were represented thanks to sensors responses and to build the model that was used to classify the samples themselves.

Finally, classification was performed, comparing two different classifiers: Partial Least Squares Discriminant Analysis (PLS-DA) and Artificial Neural Networks (ANNs). PLS-DA was successfully applied in different fields where products had to be recognized according to their place of origin or the presence of contamination, such as in milk [41], honey [42], wine [43] and cheese [44]. ANNs are complex structures that try to mimic what the human brain does. They are formed by elemental units called neurons that work like real neurons: once information arrives, they elaborate it and give an output. Each neuron is characterized by an activation function and coefficients of connectivity called weights. The overall structure is mainly composed of an input layer, hidden layers and an output layer [45]. ANNs can be used to resolve regression and classification problems, and function approximation. They found a lot of space in food applications for the analysis of data collected with electronic noses [46,47,48,49]. In this work, a feed-forward ANN trained with the Levenberg-Marquardt algorithm is used.

For PLS, dataset was split in two parts—a training set and test set—using Venetian Blinds (VB) as the cross-validation procedure. This method divides the whole dataset in j cross-validation groups; in each one, one sample is put in the test set and the others in the training set on the first step. Subsequently, in every group the sample after the previous one is taken into the test set, and the others into the training set, and so on. In this work, the number of cross-validation groups chosen was equal to 10.

For classification with PLS-DA, a toolbox made for MATLAB^®^ and released by Milano Chemometrics was used [50]. Instead, ANNs were created using the function *nntool* of the same software. This tool allows for the random splitting of the dataset in test and training sets by default.

## 3. Results and Discussion

### 3.1. GC-MS Analysis Results

**Figure 2:**
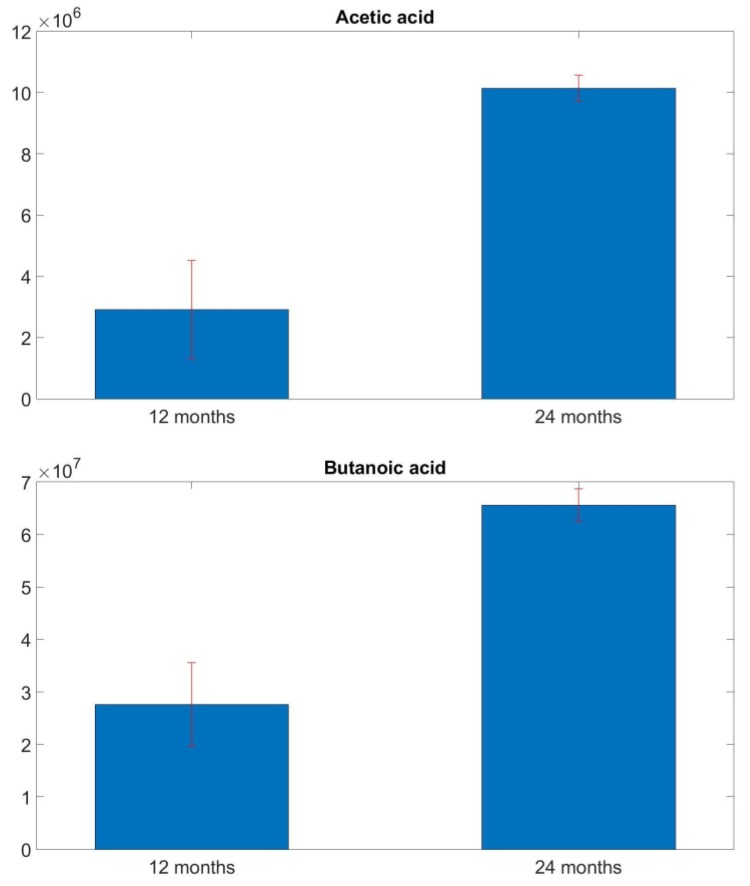

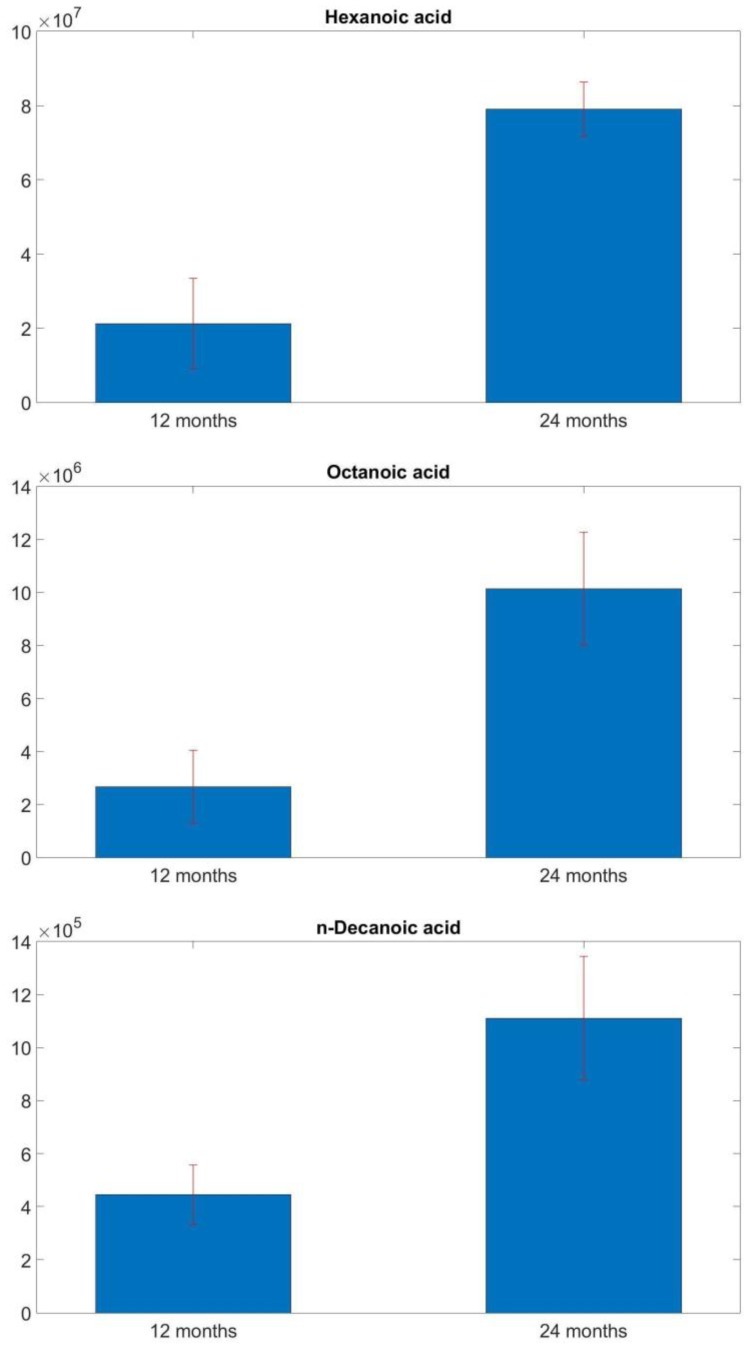
Comparison of acetic acid, butanoic acid, hexanoic acid, octanoic acid and n-decanoic acid amount between 12-months and 24-months samples. Results are presented in terms of mean ± standard deviation of the mean.

From the comparison between samples chromatograms, substantial differences were found. The main difference between 12-months and 24-months ripened grated PR lies in the amount of fatty acids that characterize this product. They are acetic acid, butanoic acid, hexanoic acid, octanoic acid, n-decanoic acid, and their presence is much greater in 24-months PR. In Figure 2, histograms for each of the aforementioned compounds are shown: results are presented in terms of mean ± standard deviation of the mean with an arbitrary unit. This result is widely confirmed in the literature. Indeed, it is well known that these fatty acids are the results of fermentation processes, especially in butter and seasoned cheese. Some studies revealed that the amount of acetic acid and butanoic acid doubles in the period between 12 and 24 months [51,52]. The same trend was observed in the other compounds, since biochemical processes that lead to their formation are very similar.

Differences were also found in comparing samples with different percentages of rind and the same rind working process; the same trend is valid both for 12-months and 24-months ripened PR. It turned out that in increasing the quantity of rind, the presence of three compounds increases. Besides butanoic and hexanoic acid, 2-nonanone has the same behavior. It is a member of the class of methyl ketones and it can be found in several foods, such as milk and cheese [53]. It is produced by the oxidative degradation of fatty acids [54]. These results suggest that both the fermentation and the degradation happen closer to the rind than in the central part of the cheese.

### 3.2. S3 Analysis Results

Once data were acquired, sensors responses were checked first. Since the first measures of each session were very different from the others, they were discarded. Consequently, there is a different number of replicas for each sample. Most likely, experimental conditions of first acquisitions were not the same as the following measures in terms of the temperature of the auto-sampler oven that the vials were put in, as explained in Section 2.3. S3 Analysis. In Table 2, a detailed description of the number of samples that were considered for the following analysis is shown.

**Figure 3:**
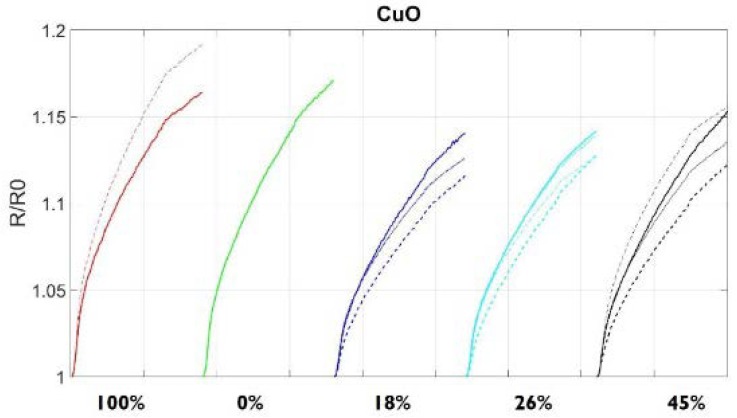

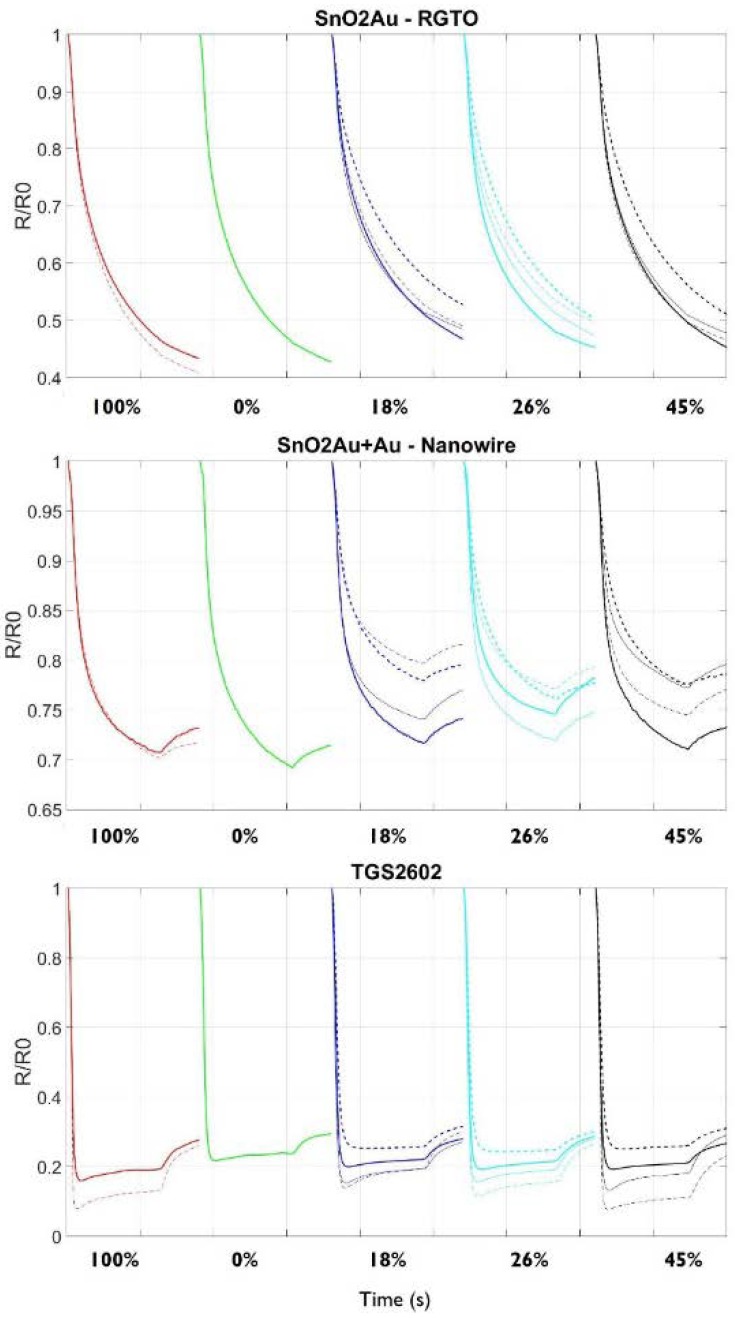
CuO, SnO_2_Au-RGTO, SnO_2_Au+Au-Nanowire and TGS2602 responses as functions of time. Samples colors: Red for 100% rind, green for 0%, blue for 18%, cyan for 26% and black for 45%. Solid line for 24-months samples and dotted line for 12-months. Finally, thicker lines represent WR and the others SR.

The choice to extract ΔR/R_0_ as a feature was made after viewing the sensors responses. In Figure 3, the resistance value, as a function of time during the injection phase, is presented for four sensors that represent the four types of MOX in the S3. They are CuO, SnO_2_Au-RGTO, SnO_2_Au+Au-Nanowire and TGS2602. Since the starting point is equal for all the measures, the variation of normalized resistance exhibit that all the sensors are capable of distinguishing samples with different concentrations of rind (samples colors: red for 100% rind, green for 0%, blue for 18%, cyan for 26% and black for 45%). In addition, they show also the ability to recognize the two ripening degrees, characterized with a solid line for 24-months samples and with a dotted line for the others. Finally, responses to the working processes are highlighted using a thicker line for WR samples as compared to SR ones.

TSG2602 and SnO_2_Au+Au nanowires seem to be the best MOX to identify ripening and rind working process at fixed concentration, since minimum resistance varies mostly for samples with the same rind percentage. Conversely, CuO and SnO_2_Au (RGTO) responses are more useful to recognize “pure” samples (0% and 100% of rind) from mixtures, since in the first case ΔR is bigger.

**Figure 4:**
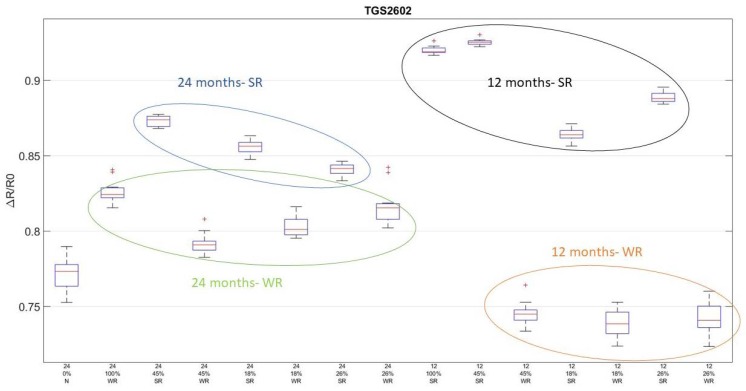
Boxplots of TGS2602 feature ΔR/R_0_. Four groups are highlighted: In blue, 24-months SR; in green, 24-months WR; in black, 12-months SR; and in orange, 12-months WR.

In Figure 4, boxplots of TGS2602 response that include ΔR/R_0_ for each sample are shown. This sensor represents the general trend that can be observed in all the sensors. Obviously, since different sensing materials are used, there are differences in the highlighted groups that overlap. On the left part of the figure, there are 24-months seasoned samples, in the upper part, grated cheese with SR, while in the lower, PR with WR. In the right part, there are 12-months ripened samples, and they follow the same trend. The first boxplot is relative to samples of 0% rind; its ΔR/R_0_ is different with respect to all the other groups, but it is more similar to WR grated PR, both at seasoning stage. This result reflects the fact that they are characterized by a greater amount of humidity.

**Figure 5:**
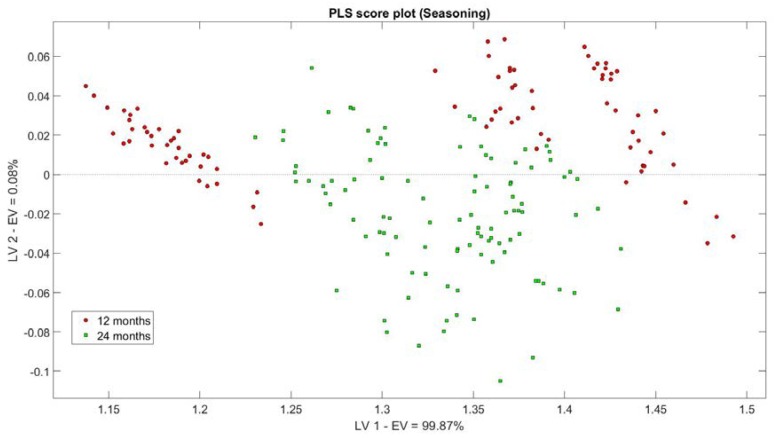
PLS score plot for all the measures divided by seasoning degree: in red circle, 12-months; in green square, 24-months. Total explained variance equal to 99.95% in first two LV.

After checking the general sensors performances, JB-test was applied to the dataset. Only 4 of the eight parameters followed a normal distribution (*p* < 0.05); they correspond to the features extracted by the two tin oxide nanowires and RGTO sensors. This was the main reason for choosing PLS. In Figure 5, PLS score plot was made, considering the first two latent variables (LV) for a total explained variance equal to 99.95% (99.87% for LV1 and 0.08% for LV2). The plot measures are divided by seasoning degree. It can be observed that the 24-months class is in the central part of the graph, while the other one is divided in the left and right part.

**Figure 6:**
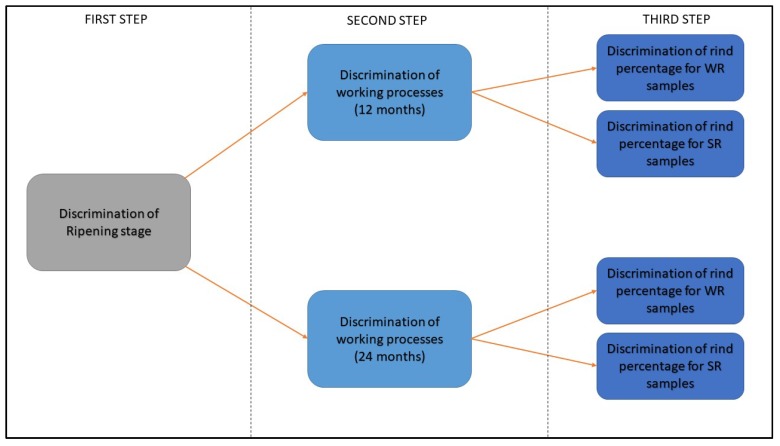
Step-by-step scheme for classification analysis.

For this reason, classification techniques were used in a hierarchical way. In addition, another motive for this choice was to simplify classification models, since this is a 15-class problem. Hence, in the first step, classifiers were used to distinguish the seasoning degree; in a second step, for each ripening state the different working processes were discriminated; finally, ring percentage was taken into account. In Figure 6, a scheme of the steps is shown.

Regarding ANNs structures, three different ones were considered, one for each step. In the first case, a two-layers architecture with 3 neurons in the input layer and 1 in the output layer was considered. For the second stage, the same number of layers was used, but two neurons were put in the first one. Finally, the third ANN had the same structure as the previous ones, but with 6 neurons in the input layer. For all the neurons, hyperbolic tangent sigmoid transfer function was chosen.

In Table 3, overall classification rate of the two classifiers is put side by side. In general, ANN classification rates are better than those of PLS-DA. Indeed, ANN is able to recognize correctly all the samples based on seasoning and rind working processes. PLS-DA performances are lower, although it can reach good classification rates. The distinction between rind percentage shows that both classifiers can classify samples with SR better than those with WR. A possible explanation for this result could be the different amount of humidity: WR samples have a higher content of humidity because of water treatment and this could cause the occupation of the adsorption sites by water molecules instead of the ones that characterize the volatile fingerprint of the samples.

To the author’s knowledge, only few researches have been carried out regarding rind composition of grated cheeses. The preparation of the samples in some works was carried out through the grating process, although the aim was to classify the different varieties of cheeses, like Swiss [55] or Emmental cheese [56]. For the latter, it has been tried unsuccessfully to find the “rind-taste” off-flavor [57]; in this case, the lack of positive results could be due to non-volatile compounds that change only the taste but not the aroma. As regards PR, cheese aroma authenticity and rind percentage recognition have been achieved with an electronic nose equipped with SnO_2_ and ZnO sensors made at SENSOR Laboratory of University of Brescia [58]. In this case, the tool was also able to distinguish samples with little differences in terms of rind percentage, such as 18% and 19%. However, unlike this study, only one type of ripening was considered (12-months) and the different working processes were not taken into consideration. Finally, the comparison with this study allows for the assessment of the utility of S3 for fraud detection, since the results point in the same direction.

## 4. Conclusions

This study aimed at verifying the possible distinction between grated PR with different rind percentages with an electronic nose, taking into account two other variables: seasoning degree and working processes of rind. In parallel, a consolidated technique, i.e., SPME GC-MS, has been used to understand which VOCs characterized analyzed samples. This combined analysis has produced promising results that pave the way to assess cheese quality and avoid frauds.

First of all, with GC-MS, the VOCs that characterize grated cheeses have been individuated. The results concerning PR are compliant with those found in the literature. Indeed, fatty acids that describe the aroma and taste profile of PR have been found in greater quantity for 24-months seasoned samples as compared to 12-months ones. In addition, VOCs, whose amount is bigger in rind compared to pulp, were found and they are acquiescent with chemical reactions that take place in this product.

The multivariate statistical analysis made with PLS indicated how to proceed during the classification stage. A hierarchical approach was used, both for PLS-DA and ANNs. ANNs classification rates are the highest, suggesting that in future they could be improved to increase their performances. These first results are encouraging, and further research is in progress to add more samples and to acquire greater statistical significance for the achieved results.

## Figures and Tables

**Table 1:** Type, composition, morphology, operating temperature, response (ΔR/R), selectivity (response ethanol/response carbon monoxide) and limit of detection (LOD) of ethanol for S3 sensors made at the SENSOR Laboratory.

Materials (Type)	Composition	Morphology	Operating Temperature (°C)	Response to 5 ppm of Ethanol	Selectivity	Limit of Detection (LOD) of Ethanol (ppm)
SnO_2_Au (n)	SnO_2_ functionalized with Au clusters	RGTO	400 °C	6.5	3	0.5
SnO_2_ (n)	SnO_2_	RGTO	300 °C	3.5	2.5	1
SnO_2_ (n)	SnO_2_	RGTO	400 °C	4	2	0.8
SnO_2_Au+Au (n)	SnO_2_ grown with Au and functionalized with gold clusters	Nanowire	350 °C	7	2.5	0.5
SnO_2_Au (n)	SnO_2_ grown with Au	Nanowire	350 °C	5	2.1	1
CuO (p)	CuO	Nanowire	400 °C	1.5	1.5	1

**Table 2:** Considered samples divided for ripening stage, rind percentage and rind working processes (WR = washed-rind, SR = scraped-rind).

SeasoningPercentage	0%	18%		26%		45%		100%	
		WR	SR	WR	SR	WR	SR	WR	SR
*12 months*	-	11	12	13	11	12	14	-	14
*24 months*	12	14	14	13	13	11	13	13	-

**Table 3:** Classification rates of Partial Least Squares Discriminant Analysis (PLS-DA) and Artificial Neural Networks (ANNs) divided per steps.

	First Step Ripening Stage	Second Step Working Processes	Third Step Rind Percentage
PLD-DA	94.7%	12 months: 100%	WR: 61.1%
			SR: 90.2%
		24 months: 79%	WR:90.2%
			SR: 95%
ANN	100%	12 months: 100%	WR: 63.8%
			SR: 96.1%
		24 months: 100%	WR: 58.8%
			SR: 100%
